# Uric acid and hyperuricemia in relation to non-alcoholic fatty liver disease among individuals with type 2 diabetes: insights from a cross-sectional analysis

**DOI:** 10.3389/fendo.2026.1720601

**Published:** 2026-03-02

**Authors:** Chunbo Li, Mengfan Zhang, Mengchun Li, Qifeng Shao

**Affiliations:** 1 Department of General Surgery, The Fifth Clinical Medical College of Henan University of Chinese Medicine (Zhengzhou People’s Hospital), Zhengzhou, China; 2 Department of Obstetrics and Gynecology, The Fifth Clinical Medical College of Henan University of Chinese Medicine (Zhengzhou People’s Hospital), Zhengzhou, China; 3 Department of Pediatric Development and Behavior, The Third Affiliated Hospital of Zhengzhou University, Zhengzhou, China

**Keywords:** hyperuricemia, non-alcoholic fatty liver disease, restricted cubic spline, type 2 diabetes mellitus, uric acid

## Abstract

**Aims:**

Considering the growing evidence linking metabolic abnormalities with liver diseases, in adults with type 2 diabetes mellitus (T2DM), this research examined the relationship between serum uric acid (UA), hyperuricemia, and non-alcoholic fatty liver disease (NAFLD) in T2DM patients.

**Methods:**

Between January 2024 and January 2025, 952 individuals with T2DM were enrolled in a single-center cross-sectional investigation conducted at the Zhengzhou People’s Hospital. Associations of UA and hyperuricemia with NAFLD were evaluated using multivariate logistic regression, accompanied by subgroup and sensitivity analyses. Restricted cubic spline (RCS) and generalized additive models (GAM) anlyses were applied to examine the non-linear dose–response association between UA and NAFLD.

**Results:**

Using the fully adjusted multivariate logistic regression model, hyperuricemia was found to be independently linked to a higher risk of NAFLD (OR 1.660, 95% CI: 1.094–2.521, *p* = 0.017). For serum UA, a rise of 1 μmol/L and a single standard deviation (SD) in its concentration corresponded to a 0.3% and 26.6% greater likelihood of developing NAFLD, respectively (OR 1.003, 95% CI: 1.001–1.005; OR 1.266, 95% CI: 1.050–1.526). Subgroup analyses revealed that hyperuricemia remained independently linked to NAFLD among individuals aged > 60 years, with hypertension, overweight/obesity, or without hyperlipidemia (*p* < 0.05). Furthermore, an elevation of one SD for UA levels was associated with a greater likelihood of NAFLD in several subgroups, including patients aged > 60 years, males, those without hyperlipidemia, and those with overweight/obesity (*p* < 0.05). Sensitivity analysis excluding patients with chronic kidney disease yielded consistent results: hyperuricemia remained significantly associated with NAFLD (OR 1.678, *p* = 0.032), and each 1-SD increase in UA was associated with a 31.6% increased risk (OR 1.316, *p* = 0.010). RCS analysis revealed a notable linear association for UA levels with the likelihood of NAFLD (*p* overall < 0.05, non-linearity *p* > 0.05). Similarly, GAM analysis further confirmed a statistically significant linear relationship between UA and NAFLD.

**Conclusion:**

Serum UA levels and hyperuricemia are independently linked to a greater likelihood of NAFLD among individuals with T2DM, underscoring the importance of UA surveillance in this group to facilitate prompt detection and precise intervention for NAFLD.

## 1 Introduction

Over recent decades, non-alcoholic fatty liver disease (NAFLD) has risen to become the leading chronic hepatic disorder globally, now estimated to affect close to one in four adults (1). Its progression can span from limited steatosis to inflammation-driven hepatic damage, severe fibrotic alteration, complete architectural distortion of the liver, and malignancies like hepatocellular carcinoma (2). Beyond its hepatic impact, NAFLD is closely intertwined with a range of metabolic abnormalities, such as type 2 diabetes mellitus (T2DM), cardiovascular disease, and chronic kidney disease (CKD) (3–5). Notably, there is a strong overlap between T2DM and NAFLD (5, 6). Studies estimate that between 50% and 70% of individuals with T2DM also have NAFLD (7, 8). The two conditions appear to reinforce one another: T2DM may accelerate the progression of NAFLD, while NAFLD can further worsen insulin resistance and impair glycemic control (9, 10). Therefore, identifying underlying contributors to NAFLD among individuals with T2DM is essential for early detection and prevention.

Uric acid (UA), the terminal metabolite of purine catabolism, has been documented to cause hyperuricemia and gout when present at elevated levels, both of which have shown a steadily increasing global prevalence (11–13). Historically linked to gout, UA is now increasingly recognized by accumulating research as having a wider involvement in the pathogenesis of metabolic syndrome, insulin resistance, cardiovascular disease, and liver disorders (14–17). Besides, previous investigations have also identified a positive relationship between UA and NAFLD within the broader population (18, 19). From a mechanistic perspective, UA may contribute to lipid deposition in hepatic tissue, oxidative stress, and inflammatory processes (20, 21). However, dedicated studies examining the relationship between UA and NAFLD specifically in individuals with T2DM remain scarce. In particular, it is unclear whether this relationship is independent of other metabolic comorbidities common in diabetes, such as obesity, hypertension, dyslipidemia, and impaired kidney function—factors that may influence both UA levels and NAFLD development.

Determining whether serum UA and hyperuricemia are independently linked to NAFLD among individuals diagnosed with T2DM holds notable clinical relevance, as it could enhance patient classification by prognostic profiles and guide focused preventive measures. Consequently, this cross-sectional analysis was designed to explore the relationship involving serum UA concentrations, hyperuricemia, plus the occurrence of NAFLD in individuals affected by T2DM, with the aim of generating evidence that may inform early detection and personalized therapeutic planning.

## 2 Methods

### 2.1 Study population

This research adopted a cross-sectional design at a single medical site, namely the Zhengzhou People’s Hospital. Eligible participants were individuals diagnosed with T2DM who had received treatment at this institution from January 2024 through January 2025. A total of 952 cases met the criteria and were incorporated into the dataset.

Entry requirements included: (1) age ≥ 18 years; (2) a confirmed diagnosis of T2DM according to established standards (22); (3) possession of full medical records containing serum UA assessments, liver ultrasound findings, and other relevant metabolic indices. Exclusion criteria comprised: (1) known liver diseases such as alcohol-related hepatic disorders, drug-induced hepatic damage, autoimmune hepatitis, viral hepatitis, or inherited metabolic liver conditions; (2) women who were pregnant or breastfeeding; (3) those with severe cerebrovascular or cardiovascular pathology, malignant tumors, or other terminal-stage illnesses; (4) serious hematologic or immune system abnormalities; (5) missing laboratory or clinical information.

Approval for the study was granted by the Ethics Committee of Zhengzhou People’s Hospital, and all procedures followed the ethical guidelines of the Declaration of Helsinki. Because the work was retrospective and data were anonymized, informed consent was waived.

### 2.2 Collection and assessment of variables

In this study, all relevant information was sourced from the hospital’s electronic health records and laboratory information management systems at the Zhengzhou People’s Hospital. Data extraction and verification were carried out by trained staff to ensure accuracy and completeness. Collected variables included demographic information (age and gender), comorbidities, anthropometric measurements, laboratory indicators, and details of medication usage. Smoking status was recorded as present if the individual was a current smoker or had a history exceeding six months. The diagnosis of T2DM was established according to the standards of the American Diabetes Association, requiring at least one diagnostic indicator—such as fasting blood glucose (FBG) greater than or equal to 7.0 mmol/L; a 2-hour plasma glucose value greater than or equal to 11.1 mmol/L obtained from an oral glucose tolerance assessment; glycated hemoglobin (HbA1c) measurement showing 6.5% or greater; or confirmed medical records indicating a prior diagnosis of diabetes or the administration of glucose-lowering treatment (22). Hypertension was determined either by a documented medical history, by systolic blood pressure (SBP) of at least 140 mmHg or diastolic blood pressure (DBP) of at least 90 mmHg, or by ongoing use of antihypertensive medications (23). Hyperlipidemia was determined based on either a documented diagnosis or abnormal lipid measurements, including triglycerides (TG) of 2.3 mmol/L or higher, total cholesterol (TC) of at least 6.2 mmol/L, or low-density lipoprotein cholesterol (LDL-C) of at least 4.1 mmol/L, as well as ongoing treatment with lipid-lowering medications (24). Excess body weight, encompassing overweight and obesity, was identified as a body mass index (BMI) of no less than 24.0 kg/m^2^, in accordance with Chinese classification standards (25). Use of antihypertensive and lipid-lowering medications was determined from documented prescriptions or treatment history in the medical records. Anthropometric data including BMI, SBP, and DBP were measured by trained healthcare professionals following standardized procedures. Laboratory parameters included liver function indicators [albumin, total bilirubin, gamma-glutamyl transferase (GGT), aspartate aminotransferase (AST), alanine aminotransferase (ALT), and alkaline phosphatase (ALP)], FBG, HbA1c, lipid profiles (high-density lipoprotein cholesterol [HDL-C], LDL-C, TC, and TG), serum UA, creatinine, and blood urea nitrogen (BUN). All tests were conducted at the time of the patient’s initial visit using standardized instruments in the hospital’s clinical laboratory.

UA levels (µmol/L) were examined both as a continuous measure and after standardization. The standardized value was derived via the z-score formula: standardized UA = (UA − mean)/standard deviation (SD), enabling evaluation of how a one-SD rise in UA relates to NAFLD likelihood. According to serum UA levels, two categories were formed: non-hyperuricemia (n = 545) and hyperuricemia (n = 407). Hyperuricemia was identified when serum UA exceeded 420 µmol/L for males or 360 µmol/L for females, according to commonly used clinical criteria in China (26).

### 2.3 Assessment and diagnosis of NAFLD

The identification of NAFLD relied on abdominal color Doppler ultrasonography carried out by skilled radiologists in accordance with uniform procedural standards. According to widely accepted international criteria (27), a diagnosis of NAFLD required meeting two conditions: (1) the presence of hepatic steatosis as indicated by typical sonographic features such as increased brightness of the hepatic parenchyma, blurring blurred intrahepatic vasculature, together with posterior beam attenuation; and (2) elimination of other potential sources of liver fat. Secondary causes were identified and excluded through a combination of medical history review, clinical records, and laboratory testing. These included high-level alcohol consumption, specified as at least 30 g/day for males and 20 g/day for females (28), persistent viral hepatitis (including types B and C), immune-mediated liver disorders, administration of drugs with fat-inducing potential (including amiodarone, methotrexate, corticosteroids), total parenteral nutrition, inflammatory bowel disease, pituitary dysfunction, hypothyroidism, and lipodystrophy.

Liver biopsy was not performed for this research; NAFLD was diagnosed exclusively through imaging findings. Furthermore, patients were not subclassified into simple steatosis or non-alcoholic steatohepatitis (NASH). To ensure diagnostic consistency, all ultrasound images were independently evaluated by two radiologists. In cases of disagreement, a third senior radiologist reviewed the scans to make the final judgment. Based on the presence or absence of NAFLD, participants were categorized as either non-NAFLD or NAFLD.

### 2.4 Statistical procedures

Data processing and analysis were carried out using R software (v4.4.3; R Foundation for Statistical Computing, Vienna, Austria). The Shapiro–Wilk procedure evaluated whether continuous variables followed a normal distribution. As the data deviated from normality, they were summarized using medians and interquartile ranges (IQRs). Categorical data were summarized as frequency counts with corresponding percentages. For continuous variables not conforming to normality, intergroup differences were assessed using the Mann–Whitney U method, whereas categorical variables were compared through the Chi-square test or, when applicable, Fisher’s exact approach.

Covariates included in the multivariable models were selected based on statistical significance (*p* < 0.05) in univariable logistic regression analyses. Multivariable logistic regression was applied to examine the associations among serum UA concentrations, hyperuricemia, and NAFLD. NAFLD served as the outcome variable, while UA was analyzed both as a continuous measure and as a Z-score–standardized value, and hyperuricemia was included as a binary predictor. Results were expressed as odds ratios (ORs) with 95% confidence intervals (CIs), obtained from three separately specified regression models. The first model (Model 1) included no adjustments; the second (Model 2) controlled solely for age; and the third (Model 3) incorporated an extensive range of covariates—such as age, hypertension, hyperlipidemia, overweight/obesity, BMI, DBP, ALT, AST, total bilirubin, ALP, GGT, albumin, FBG, TC, TG, HDL-C, LDL-C, serum creatinine, and BUN. Besides, analyses were stratified according to subgroups defined by demographic and clinical characteristics, including age, gender, hypertension, hyperlipidemia, and overweight or obesity. To further verify the stability of the findings, cases involving CKD were excluded for a separate sensitivity assessment.

Additionally, the non-linear dose–response association between UA levels and NAFLD risk was examined and visualized using restricted cubic spline (RCS) regression and generalized additive models (GAM). For RCS, *p*-values for the overall association (*p*-overall) and non-linearity (*p*-nonlinear) were calculated to determine whether the relationship was linear or non-linear. For GAM, the effective degrees of freedom (EDF) were used to assess linearity, with values close to 1 indicating a linear relationship, and corresponding *p*-values were reported to evaluate statistical significance. All statistical analyses were conducted using two-tailed tests, with a *p*-value below 0.05 indicating statistical significance.

## 3 Results

### 3.1 Clinical characteristics

As shown in **Table 1**, this study included 952 individuals diagnosed with T2DM, whose median age was 67 years (IQR: 59–72). Among them, 407 individuals (42.8%) were diagnosed with hyperuricemia. Relative to their counterparts without the condition, individuals belonging to the hyperuricemia group exhibited a notably greater occurrence of hypertension (80.2% vs. 70.8%), hyperlipidemia (44.4% vs. 32.4%), overweight or obesity (65.8% vs. 46.3%), and NAFLD (54.5% vs. 39.9%), and demonstrated a higher likelihood of undergoing antihypertensive therapy (59.9% vs. 49.0%). Additionally, individuals with hyperuricemia showed significantly higher values for BMI, GGT, triglycerides, TC, LDL-C, BUN, creatinine, and UA levels, while their HDL-C levels were notably lower (all *p* < 0.05). Conversely, the two groups showed no significant statistical variation regarding age, gender distribution, smoking status, use of lipid-lowering agents, SBP, DBP, ALT, AST, total bilirubin, ALP, albumin, FBG, or HbA1c (all *p* > 0.05).

**Table 1:** Clinical characteristics grouped by hyperuricemia status.

Variables	Total population	Non-hyperuricemia	Hyperuricemia	*p* value
N	952	765	187	
Age, years	67.00 (59.0, 72.0)	67.00 (59.0, 72.0)	66.00 (57.0, 73.0)	0.910
Gender, n (%)				0.193
Male	519 (54.5)	425 (55.6)	94 (50.3)	
Female	433 (45.5)	340 (44.4)	93 (49.7)	
Smoking, n (%)	122 (12.8)	98 (12.8)	24 (12.8)	0.993
Hypertension, n (%)	692 (72.7)	542 (70.8)	150 (80.2)	0.010
Antihypertensive medications, n (%)	487 (51.2)	375 (49.0)	112 (59.9)	0.008
Hyperlipidemia, n (%)	331 (34.8)	248 (32.4)	83 (44.4)	0.002
Lipid-lowering agents, n (%)	198 (20.8)	161 (21.0)	37 (19.8)	0.704
Overweight/obesity, n (%)	477 (50.1)	354 (46.3)	123 (65.8)	< 0.001
NAFLD, n (%)	407 (42.8)	305 (39.9)	102 (54.5)	< 0.001
BMI, kg/m^2^	24.0 (22.1, 26.3)	23.7 (21.8, 26.1)	25.0 (23.2, 27.7)	< 0.001
SBP, mmHg	135.0 (124.0, 149.0)	135.0 (124.0, 149.0)	135.0 (124.0, 148.0)	0.685
DBP, mmHg	80.0 (73.0, 88.0)	80.0 (73.0, 88.0)	80.0 (73.0, 89.0)	0.956
ALT, U/L	18.2 (12.9, 27.1)	17.9 (12.9, 26.6)	19.2 (12.5, 29.8)	0.240
AST, U/L	17.3 (14.2, 22.4)	17.2 (14.3, 21.9)	17.7 (14.0, 23.7)	0.185
Total bilirubin, µmol/L	9.4 (7.1, 12.4)	9.2 (7.3, 12.3)	9.6 (6.9, 12.4)	0.986
ALP, U/L	69.6 (57.3, 84.9)	69.0 (57.4, 84.8)	73.4 (57.3, 87.4)	0.184
GGT, U/L	21.1 (14.3, 33.3)	20.1 (14.0, 32.5)	24.6 (16.2, 38.9)	< 0.001
Albumin, g/L	41.1 (39.0, 43.3)	41.0 (38.8, 43.2)	41.4 (39.4, 43.8)	0.190
FBG, mmol/L	6.6 (5.4, 8.2)	6.6 (5.4, 8.2)	6.5 (5.4, 7.9)	0.472
HbA1c, %	8.4 (7.1, 10.0)	8.5 (7.1, 10.2)	8.3 (7.0, 9.5)	0.062
Triglycerides, mmol/L	1.4 (1.0, 2.0)	1.3 (0.9, 1.8)	1.8 (1.3, 2.5)	< 0.001
Total cholesterol, mmol/L	4.3 (3.6, 5.0)	4.2 (3.5, 4.9)	4.6 (3.8, 5.4)	< 0.001
LDL-C, mmol/L	2.4 (1.8, 3.1)	2.4 (1.8, 3.0)	2.7 (2.1, 3.4)	< 0.001
HDL-C, mmol/L	1.2 (1.0, 1.4)	1.2 (1.0, 1.4)	1.1 (1.0, 1.3)	0.034
BUN, mmol/L	5.7 (4.6, 7.0)	5.5 (4.6, 6.8)	6.3 (5.0, 8.8)	< 0.001
Creatinine, μmol/L	69.9 (57.9, 84.8)	67.3 (56.1, 81.7)	82.7 (66.1, 111.1)	< 0.001
UA, μmol/L	314.0 (257.0, 371.0)	291.0 (245.0, 333.0)	438.0 (393.0, 481.0)	< 0.001

NAFLD, non-alcoholic fatty liver disease; BMI, body mass index; SBP, systolic blood pressure; DBP, diastolic blood pressure; ALT, alanine aminotransferase; AST, aspartate aminotransferase; ALP, alkaline phosphatase; GGT, gamma-glutamyl transferase; FBG, fasting blood glucose; HbA1c, glycated hemoglobin A1c; LDL-C, low-density lipoprotein-cholesterol; HDL-C, high-density lipoprotein-cholesterol; BUN, blood urea nitrogen; UA, uric acid.

In addition, as shown in **Table 2**, participants were categorized as either non-NAFLD (n = 545) or NAFLD (n = 407). Compared with the non-NAFLD group, patients in the NAFLD group exhibited significantly higher levels of multiple indicators, including the prevalence of hypertension, hyperlipidemia, and overweight/obesity, BMI and DBP, as well as serum ALT, AST, total bilirubin, GGT, albumin, FBG, HbA1c, triglycerides, TC, LDL-C, and serum UA (all *p* < 0.05). In contrast, patients with NAFLD were significantly younger and had lower levels of HDL-C, BUN, and serum creatinine compared with those without NAFLD (all *p* < 0.05), while no significant differences were observed between the two groups in terms of sex distribution, smoking status, use of antihypertensive or lipid-lowering medications, SBP, or ALP levels (all *p* > 0.05).

**Table 2:** Clinical characteristics grouped by NAFLD status.

Variables	Non-NAFLD	NAFLD	*p* value
N	545	407	
Age, years	68.0 (61.0, 73.0)	65.0 (55.0, 71.0)	< 0.001
Gender, n (%)			0.704
Male	300 (55.0)	219 (53.8)	
Female	245 (45.0)	188 (46.2)	
Smoking, n (%)	69 (12.7)	53 (13.0)	0.869
Hypertension, n (%)	373 (68.4)	319 (78.4)	0.001
Antihypertensive medications, n (%)	266 (48.8)	221 (54.3)	0.094
Hyperlipidemia, n (%)	146 (26.8)	185 (45.5)	< 0.001
Lipid-lowering agents, n (%)	121 (22.2)	77 (18.9)	0.217
Overweight/obesity, n (%)	183 (33.6)	294 (72.2)	< 0.001
BMI, kg/m^2^	22.8 (21.0, 24.9)	25.8 (23.8, 27.8)	< 0.001
SBP, mmHg	135.0 (123.0, 149.0)	136.0 (125.0, 150.0)	0.082
DBP, mmHg	79.0 (72.0, 87.0)	81.0 (74.0, 88.0)	0.001
ALT, U/L	15.3 (11.8, 21.2)	23.6 (16.0, 35.6)	< 0.001
AST, U/L	16.2 (13.6, 20.2)	19.4 (15.1, 25.4)	< 0.001
Total bilirubin, µmol/L	8.9 (6.7, 11.7)	10.0 (7.8, 13.0)	< 0.001
ALP, U/L	70.9 (57.7, 86.9)	68.7 (56.5, 82.5)	0.098
GGT, U/L	17.7 (12.5, 26.6)	27.1 (17.9, 43.6)	< 0.001
Albumin, g/L	40.4 (38.2, 42.8)	41.7 (39.8, 43.8)	< 0.001
FBG, mmol/L	6.3 (5.1, 7.8)	6.9 (5.8, 8.5)	< 0.001
HbA1c, %	8.3 (7.0, 10.0)	8.5 (7.4, 10.1)	0.044
Triglycerides, mmol/L	1.2 (0.9, 1.6)	1.6 (1.2, 2.4)	< 0.001
Total cholesterol, mmol/L	4.2 (3.4, 4.9)	4.5 (3.7, 5.1)	0.001
LDL-C, mmol/L	2.3 (1.8, 3.0)	2.6 (1.9, 3.2)	0.001
HDL-C, mmol/L	1.2 (1.0, 1.4)	1.1 (1.0, 1.3)	< 0.001
BUN, mmol/L	5.8 (4.8, 7.3)	5.5 (4.5, 6.7)	0.002
Creatinine, μmol/L	71.3 (57.4, 86.7)	67.5 (58.3, 81.7)	0.047
UA, μmol/L	303.0 (245.0, 359.0)	325.0 (272.0, 388.0)	< 0.001

NAFLD, non-alcoholic fatty liver disease; BMI, body mass index; SBP, systolic blood pressure; DBP, diastolic blood pressure; ALT, alanine aminotransferase; AST, aspartate aminotransferase; ALP, alkaline phosphatase; GGT, gamma-glutamyl transferase; FBG, fasting blood glucose; HbA1c, glycated hemoglobin A1c; LDL-C, low-density lipoprotein-cholesterol; HDL-C, high-density lipoprotein-cholesterol; BUN, blood urea nitrogen; UA, uric acid.

### 3.2 Multivariate association of UA and hyperuricemia with NAFLD

As presented in **Table 3**, serum UA concentrations and the presence of hyperuricemia were both significantly correlated with the likelihood of developing NAFLD in individuals diagnosed with T2DM. In the unadjusted model (Model 1), the OR for NAFLD in participants with hyperuricemia was 1.810 (95% CI: 1.311–2.498, *p* < 0.001), indicating a 1.81-fold greater probability of disease occurrence compared with those free of hyperuricemia. And a 1 μmol/L elevation in serum UA was associated with an OR of 1.004 (95% CI: 1.002–1.005, *p* < 0.001) for NAFLD, corresponding to a 0.4% increase in risk. Additionally, each 1-SD rise in UA was linked to an OR of 1.395 (95% CI: 1.221–1.594, *p* < 0.001), representing a 39.5% higher likelihood of NAFLD.

**Table 3:** Multivariate association of UA and hyperuricemia with NAFLD.

Variables	Model 1		Model 2		Model 3	
	OR (95% CI)	*p* value	OR (95% CI)	*p* value	OR (95% CI)	*p* value
Hyperuricemia						
No	Ref		Ref		Ref	
Yes	1.810 (1.311, 2.498)	< 0.001	1.779 (1.279, 2.474)	0.001	1.660 (1.094, 2.521)	0.017
UA	1.004 (1.002, 1.005)	< 0.001	1.003 (1.002, 1.005)	< 0.001	1.003 (1.001, 1.005)	0.014
Standardized UA	1.395 (1.221, 1.594)	< 0.001	1.327 (1.158, 1.521)	< 0.001	1.266 (1.050, 1.526)	0.014

UA, uric acid; NAFLD, non-alcoholic fatty liver disease; OR, odds ratio; CI, confidence interval.

Model 1: Unadjusted for covariates; Model 2: Adjusted for age only; Model 3: Adjusted for age, hypertension, hyperlipidemia, overweight/obesity, body mass index, diastolic blood pressure, alanine aminotransferase, aspartate aminotransferase, total bilirubin, alkaline phosphatase, gamma-glutamyl transferase, albumin, fasting blood glucose, triglycerides, total cholesterol, low-density lipoprotein cholesterol, high-density lipoprotein cholesterol, blood urea nitrogen, and creatinine.

After adjusting for age alone (Model 2), the associations remained significant. Hyperuricemia was linked to a 1.78-fold higher risk of NAFLD (OR = 1.779, *p* = 0.001). Additionally, per 1 μmol/L rise of UA, the risk rose by approximately 0.3% (OR = 1.003, *p* < 0.001), while a 1-SD increment of UA corresponded to a 32.7% higher risk (OR = 1.327, *p* < 0.001).

After full adjustment in Model 3—accounting for demographic, metabolic, and biochemical covariates—hyperuricemia remained an independent determinant of NAFLD, with an OR of 1.660 (95% CI: 1.094–2.521, *p* = 0.017). Additionally, each 1 μmol/L rise in UA corresponded to an OR of 1.003 for NAFLD (95% CI: 1.001–1.005, *p* = 0.014), while a one-SD increment was associated with a 26.6% higher risk (OR = 1.266, 95% CI: 1.050–1.526, *p* = 0.014).

### 3.3 Subgroup analysis

**Table 4** presented findings from a multivariate adjustment–based subgroup evaluation, which examined variations in the links between serum UA, hyperuricemia, and NAFLD across distinct population categories.

**Table 4:** Multivariate subgroup analysis of uric acid and hyperuricemia in relation to NAFLD.

Subgroups	Hyperuricemia		UA		Standardized UA	
	OR (95% CI)	*p* value	OR (95% CI)	*p* value	OR (95% CI)	*p* value
Age						
≤ 60 years	1.522 (0.672, 3.444)	0.314	1.004 (1.000, 1.008)	0.071	1.387 (0.973, 1.978)	0.071
> 60 years	1.906 (1.135, 3.200)	0.015	1.003 (1.000, 1.005)	0.032	1.292 (1.022, 1.634)	0.032
Gender						
Male	1.672 (0.878, 3.182)	0.118	1.004 (1.001, 1.006)	0.013	1.376 (1.071, 1.769)	0.013
Female	1.561 (0.841, 2.897)	0.158	1.002 (0.998, 1.005)	0.310	1.167 (0.866, 1.571)	0.310
Hypertension						
Yes	1.728 (1.065, 2.804)	0.027	1.002 (1.000, 1.005)	0.067	1.237 (0.986, 1.551)	0.067
No	1.700 (0.632, 4.571)	0.293	1.004 (0.999, 1.008)	0.103	1.373 (0.938, 2.008)	0.103
Hyperlipidemia						
Yes	1.098 (0.544, 2.213)	0.795	1.000 (0.996, 1.003)	0.846	0.968 (0.696, 1.345)	0.846
No	2.056 (1.178, 3.589)	0.011	1.005 (1.002, 1.007)	0.001	1.508 (1.180, 1.926)	0.001
Overweight/obesity						
Yes	1.833 (1.055, 3.185)	0.032	1.003 (1.000, 1.005)	0.038	1.267 (1.013, 1.584)	0.038
No	1.555 (0.748, 3.231)	0.237	1.003 (0.999, 1.007)	0.145	1.287 (0.917, 1.806)	0.145

UA, uric acid; NAFLD, non-alcoholic fatty liver disease; OR, odds ratio; CI, confidence interval.

The subgroup analysis adjusted for age, hypertension, hyperlipidemia, overweight/obesity, body mass index, diastolic blood pressure, alanine aminotransferase, aspartate aminotransferase, total bilirubin, alkaline phosphatase, gamma-glutamyl transferase, albumin, fasting blood glucose, triglycerides, total cholesterol, low-density lipoprotein cholesterol, high-density lipoprotein cholesterol, blood urea nitrogen, and creatinine.

In the age-stratified analysis, using 60 years as a clinically and epidemiologically meaningful cutoff, individuals aged above 60 years exhibited a significant association between hyperuricemia and NAFLD (OR = 1.906, 95% CI: 1.135–3.200, *p* = 0.015), whereas no such relationship was observed in participants aged 60 years or below (*p* = 0.314). Among those over 60, each 1 μmol/L rise of UA corresponded to an approximately 0.3% increase in NAFLD risk (*p* = 0.032), while a one-SD increment of UA corresponded with a 29.2% greater likelihood of NAFLD (OR = 1.292, *p* = 0.032).

In the subgroup analysis for male patients, a 1 μmol/L elevation of UA was linked to a 0.4% rise in NAFLD risk (*p* = 0.013), while a one-SD increment of UA corresponded to a 37.6% greater likelihood (OR = 1.376, *p* = 0.013). However, in female participants, analyses revealed no evidence of a statistically significant association (all *p* > 0.05).

In participants with hypertension, a significant association was observed between hyperuricemia and NAFLD (OR = 1.728, *p* = 0.027), whereas this association was not significant among those without hypertension (*p* = 0.293). Similarly, in the hypertensive subgroup, each 1-SD increase in UA was associated with an estimated 23.7% higher NAFLD risk, though this was only marginally significant (*p* = 0.067).

In the subgroup without hyperlipidemia, the associations were strongest: hyperuricemia was significantly associated with a twofold elevation for NAFLD risk (OR = 2.056, *p* = 0.011); each 1 μmol/L rise of UA corresponded to a 0.5% higher risk (*p* = 0.001); and each 1-SD increment of UA conferred a 50.8% greater risk (OR = 1.508, *p* = 0.001). These associations were not statistically significant in individuals with hyperlipidemia (all *p* > 0.05).

Among overweight or obese individuals, a notable link was observed between hyperuricemia and NAFLD (OR = 1.833, *p* = 0.032). And a rise of 1 μmol/L of UA corresponded to an estimated 0.3% elevation for NAFLD risk (*p* = 0.038), while a 1-SD increment of UA was related to a 26.7% higher likelihood of NAFLD (*p* = 0.038).

### 3.4 Multivariate association of UA and hyperuricemia with NAFLD after excluding patients with CKD

As shown in **Table 5**, a multivariate analysis excluding individuals with CKD was performed to reduce potential confounding. Findings indicated that both serum UA concentrations and hyperuricemia retained a significant association with NAFLD despite this exclusion. Within the unadjusted Model 1, hyperuricemic patients exhibited a 2.56-fold elevated likelihood of NAFLD relative to non-hyperuricemic individuals (OR = 2.560, 95% CI: 1.719–3.815, *p* < 0.001). A rise of 1 μmol/L of UA corresponded to an estimated 0.6% higher risk (OR = 1.006, *p* < 0.001), while a one-SD increment of UA was linked to a 70.5% increase in risk (OR = 1.705, *p* < 0.001).

**Table 5:** Multivariate association of uric acid and hyperuricemia with NAFLD after excluding patients with CKD.

Variables	Model 1		Model 2		Model 3	
	OR (95% CI)	*p* value	OR (95% CI)	*p* value	OR (95% CI)	*p* value
Hyperuricemia						
No	Ref		Ref		Ref	
Yes	2.560 (1.719, 3.815)	< 0.001	2.441 (1.624, 3.668)	< 0.001	1.678 (1.045, 2.694)	0.032
UA	1.006 (1.004, 1.008)	< 0.001	1.005 (1.003, 1.007)	< 0.001	1.003 (1.001, 1.005)	0.010
Standardized UA	1.705 (1.449, 2.006)	< 0.001	1.602 (1.356, 1.891)	< 0.001	1.316 (1.067, 1.624)	0.010

UA, uric acid; NAFLD, non-alcoholic fatty liver disease; CKD, chronic kidney disease; OR, odds ratio; CI, confidence interval.

Model 1: Unadjusted for covariates; Model 2: Adjusted for age only; Model 3: Adjusted for age, hypertension, hyperlipidemia, overweight/obesity, body mass index, diastolic blood pressure, alanine aminotransferase, aspartate aminotransferase, total bilirubin, alkaline phosphatase, gamma-glutamyl transferase, albumin, fasting blood glucose, triglycerides, total cholesterol, low-density lipoprotein cholesterol, high-density lipoprotein cholesterol, blood urea nitrogen, and creatinine.

Within the age-adjusted Model 2, hyperuricemia remained linked to a 2.44-fold elevation in NAFLD risk (OR = 2.441, *p* < 0.001). A rise of 1 μmol/L of UA was related to an estimated 0.5% greater risk (OR = 1.005, *p* < 0.001), whereas a one-SD increment of UA corresponded to a 60.2% increase in risk (OR = 1.602, *p* < 0.001). After adjusting for covariates with *p* < 0.05 in univariate analysis (Model 3), hyperuricemia maintained a significant association with NAFLD, with an OR of 1.678, and a 95% CI ranging from 1.045 to 2.694 (*p* = 0.032). Additionally, a rise of 1 μmol/L of UA was linked to a 0.3% increase in NAFLD risk (*p* = 0.010), and each 1-SD increase in UA corresponded to a 31.6% increase in risk (OR = 1.316, *p* = 0.010).

### 3.5 RCS and GAM analyses: linear pattern linking UA to NAFLD

**Figure 1:**
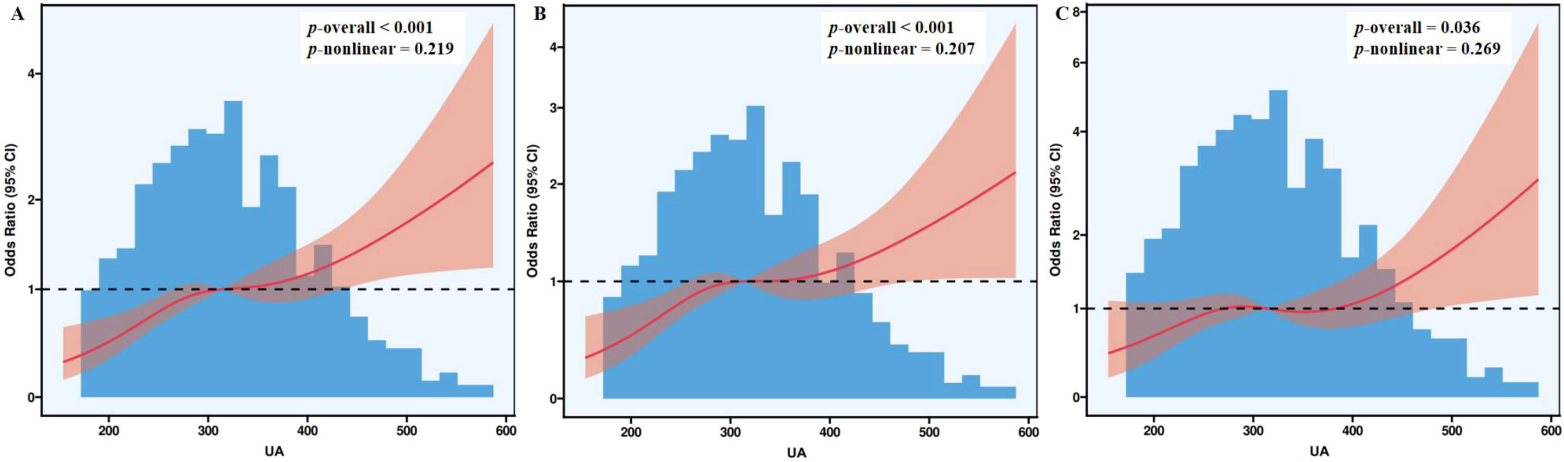
The RCS plots of uric acid and NAFLD in models 1 **(A)**, 2 **(B)**, and 3 **(C)**. **(A)** Model 1, unadjusted; **(B)** Model 2, adjusted for age; and **(C)** Model 3, adjusted for age, hypertension, hyperlipidemia, overweight/obesity, body mass index, diastolic blood pressure, alanine aminotransferase, aspartate aminotransferase, total bilirubin, alkaline phosphatase, gamma-glutamyl transferase, albumin, fasting blood glucose, total cholesterol, triglycerides, high-density lipoprotein cholesterol, low-density lipoprotein cholesterol, creatinine, and blood urea nitrogen. RCS, restricted cubic spline; UA, uric acid; CI, confidence interval.

**Figure 1** showed that, in RCS regression, higher serum UA levels corresponded to a steady rise in NAFLD risk across three analytical models—unadjusted, age-adjusted, and fully adjusted for other variables. All models reached statistical significance (*p* < 0.001 for the first two; *p* = 0.036 for the third), and the absence of non-linearity (*p* > 0.05) indicated a consistent linear trend.

**Figure 2:**
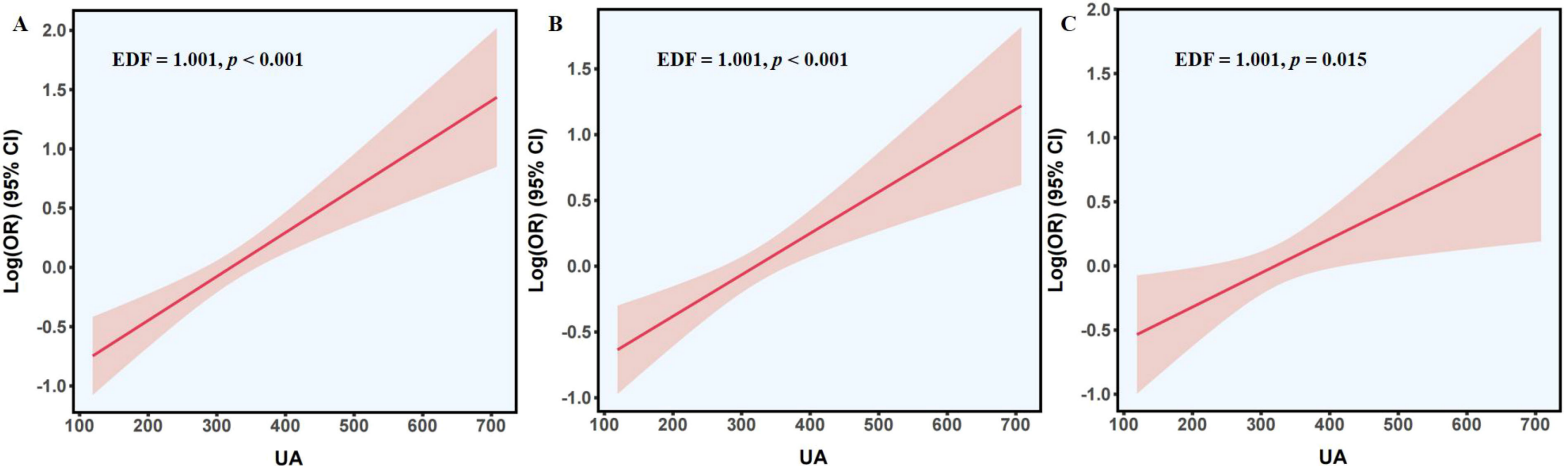
The GAM plots of uric acid and NAFLD in models 1 **(A)**, 2 **(B)**, and 3 **(C)**. GAM, generalized additive model; UA, uric acid; NAFLD, non-alcoholic fatty liver disease; OR, odds ratio; CI, confidence interval; EDF, effective degrees of freedom.

Consistently, GAM analyses (**Figure 2**) yielded EDF values close to 1 in all models (EDF = 1.001), further supporting a linear association between UA and NAFLD, with all corresponding P-values indicating statistical significance.

## 4 Discussion

This study, conducted as a single-center cross-sectional analysis, investigated the association among serum UA, hyperuricemia, and NAFLD among people diagnosed with T2DM. Upon controlling for various metabolic variables, including age, obesity, hypertension, and dyslipidemia, the multivariable logistic regression analysis revealed that hyperuricemia remained a standalone contributor to the risk of NAFLD. Additionally, for every SD elevation in UA levels, the odds of NAFLD were observed to increase by approximately 26.6%. Subgroup analyses indicated that this association was particularly evident among patients aged over 60, those with hypertension, individuals with excess body weight, as well as those lacking. Within such subgroups, standardized UA also showed a consistent association with NAFLD risk. The robustness of these findings was further supported by sensitivity analyses excluding patients with CKD. Moreover, RCS and GAM analyses revealed an evident proportional dose-response pattern linking UA concentrations to NAFLD risk. Taken together, these findings suggest that serum UA may function as a practical and economical indicator for supporting early identification and classification of NAFLD in patients with T2DM.

Over the past few years, a substantial body of research has revealed a significant association between serum UA, hyperuricemia, and NAFLD in the general population. However, whether this association holds independently in high-risk metabolic groups such as individuals with T2DM remains unclear. Existing literature has shown that hyperuricemia and NAFLD share overlapping epidemiological features, pathophysiological mechanisms, and clinical manifestations, suggesting an intrinsic link worth further exploration. To begin with, a large number of findings from both observational and forward-looking investigations indicate that elevated UA concentrations show a positive relationship in terms of the prevalence and severity of NAFLD, as well as its occurrence (29). A recent comprehensive review and pooled analysis confirmed that individuals with hyperuricemia have a markedly greater likelihood of developing NAFLD relative to individuals exhibiting normal UA levels, with this trend particularly pronounced in Asian populations (30). Both men and women exhibit increased risk, although the magnitude appears more substantial in females, implying that gender may be a potential effect modifier (30). In addition, some evidence suggests that NAFLD itself may contribute to increased UA levels, supporting the possibility of a bidirectional relationship (29). While causality remains to be established, this reciprocal pattern reflects the complex interplay between the two conditions within the broader context of metabolic dysfunction. Moreover, hyperuricemia has been associated with more severe hepatic impairment in NAFLD patients (31). These individuals often show pronounced steatosis and inflammatory changes on imaging or laboratory evaluation, especially in male patients (31). Mechanistically, elevated UA may exacerbate hepatic lipid accumulation and inflammation through various pathways, including enhanced oxidative stress, inhibition of the SIRT1 signaling axis, activation of NF-κB-mediated inflammatory cascades, and promotion of insulin resistance (31). Additionally, sex-specific differences appear to influence the pathogenic process. Animal studies have demonstrated that male rats with hyperuricemia develop more severe hepatic steatosis and inflammation, which may be linked to decreased androgen levels, downregulated SIRT1 expression, and reduced FOXO3a phosphorylation—suggesting that sex hormones might mediate UA-induced hepatic injury (31). Clinically, a retrospective study reported that hyperuricemia becomes more common alongside increasing NAFLD severity, reaching nearly 60% in advanced cases (32). Furthermore, NAFLD patients with concurrent hyperuricemia are more likely to present with other metabolic comorbidities such as hypertension, excess body weight, and diabetes, underscoring the contribution of UA as a synergistic factor in NAFLD progression (32). These data suggest that hyperuricemia represents both a notable contributor to the initiation of NAFLD and a potential indicator of disease severity. Future studies should aim to clarify the causal mechanisms linking UA and NAFLD, and investigate whether interventions targeting uric acid metabolism can mitigate or delay NAFLD progression, thereby offering new opportunities for prevention and treatment.

Besides, a growing body of evidence from both observational and long-term follow-up studies has consistently demonstrated that elevated serum UA shows a notable link to an elevated likelihood of developing NAFLD. As an example, a large-scale investigation performed in a Chinese cohort revealed that each SD increase in UA corresponded to a 40% rise in the one-year incidence of NAFLD among individuals without baseline liver disease (33). In comparison, our study, which exclusively focused on patients with established T2DM, observed a relatively lower but still statistically significant effect size, with each one-SD increase in serum UA associated with an approximately 26.6% higher odds of NAFLD. This difference in magnitude may be partly explained by variations in study population characteristics, as patients with T2DM already exhibit a higher baseline metabolic risk and a greater prevalence of NAFLD, potentially attenuating the relative contribution of UA compared with that observed in the general population. Similarly, Sun et al., through a meta-analysis encompassing over two million participants, found that participants exhibiting elevated UA concentrations showed an 88% greater likelihood of developing NAFLD relative to participants who exhibited lower measurements, while the observed relationship persisted across various population subsets (34). The stronger association reported in this meta-analysis may also reflect differences in study design (predominantly prospective cohorts), NAFLD ascertainment methods, and broader population inclusion, whereas our cross-sectional analysis provides complementary evidence specifically within a high-risk diabetic population. Notably, the predictive role of UA appears particularly prominent in non-obese populations. A study targeting postmenopausal women showed that even among those with normal body weight, elevated UA levels were strongly linked to increased NAFLD prevalence, indicating that this relationship may not be entirely mediated by obesity (35). Building on previous evidence, our subgroup analyses demonstrated that the association between UA and NAFLD was significant both among individuals without hyperlipidemia and those with overweight or obesity. Notably, the association appeared stronger in participants without hyperlipidemia, suggesting that the UA-NAFLD relationship is not solely explained by traditional metabolic abnormalities and may operate through additional pathways beyond excess body weight or dyslipidemia. Beyond static measurements, the trajectory of UA over time is also relevant. Through a forward-looking cohort investigation, Ma and colleagues observed that individuals with persistently rising UA levels exhibited an incremental rise in NAFLD likelihood corresponding to dosage levels, wherein the group with the greatest trajectory displayed more than twice the incidence compared with the lowest (36). Mechanistically, UA may contribute to hepatic fat accumulation and liver injury by promoting oxidative stress, activating the NLRP3 inflammasome, and exacerbating insulin resistance (37). However, whether this relationship is causal remains controversial. Mendelian randomization studies by Li et al. (2022) and Tang et al. (2022) suggest that while NAFLD can raise UA levels, genetically higher UA does not appear to causally increase NAFLD risk (38, 39). These findings suggest that serum UA may function primarily as a marker reflecting underlying metabolic dysfunction rather than a direct etiological driver of NAFLD, particularly in metabolically complex populations such as patients with T2DM. Subgroup analyses further emphasize that the association of UA with NAFLD appears more pronounced in non-obese and even underweight individuals. He et al. (2025) reported that among normal or lean participants, elevated UA was independently associated with NAFLD, accounting for up to 17% of the disease risk (40). This challenges the traditional view that NAFLD is predominantly an obesity-related condition. Moreover, specific populations such as postmenopausal women and younger adults exhibit distinct patterns in the UA-NAFLD relationship, emphasizing the importance of considering population heterogeneity in future research. Clinically, UA has shown potential as an indicator associated with the initiation and advancement of NAFLD. Besides, according to the findings of Zhou et al., the integration of UA measurements with serum ferritin assessment enhanced the accuracy of NAFLD diagnosis (41). Preliminary evidence also suggests that UA-lowering therapies may benefit NAFLD management, though robust data from large-scale interventional trials are still lacking (37). In summary, while elevated UA is clearly correlated with NAFLD and may serve as a predictive indicator, current evidence does not confirm a causal role. Future research should aim to clarify its mechanistic contribution in various subpopulations, identify its specific role in disease progression, and evaluate the true therapeutic value of lowering UA in the context of NAFLD prevention and treatment.

UA may function not only as an indicator of metabolic impairment but also as an active driver initiating and advancing NAFLD. One hypothesized pathway suggests that elevated UA can impair mitochondrial function in hepatocytes, leading to reduced fatty acid oxidation and increased intracellular triglyceride accumulation, ultimately promoting hepatic steatosis (20). In parallel, UA is capable of generating reactive oxygen species, a key contributor to oxidative stress and subsequent damage to hepatocytes (42). Additionally, UA has been reported to trigger NLRP3 inflammasome activation, promoting the secretion of pro-inflammatory mediators including TNF-α and IL-6, thereby fostering a persistent mild inflammatory state—an important determinant in the progression from NAFLD to NASH (21, 43, 44). Together, these interrelated pathways suggest that UA might contribute to the pathogenesis of NAFLD while potentially serving as a target for therapeutic intervention.

Compared to previous studies, the present investigation offers several key strengths and innovations. Most importantly, this study directly quantifies and contextualizes the effect size of serum UA within a T2DM-specific population, thereby addressing a gap in the existing literature that has largely focused on the general population. To begin with, the study included a comparatively substantial number of participants, all of whom were enrolled in an authentic clinical environment, thereby enhancing the generalizability of the findings. Second, the analysis accounted for factors that could influence the outcomes—such as hypertension, lipid metabolism disorders, and excess body weight—thereby enhancing the robustness and reliability of the regression models. Third, UA was assessed in forms of both continuous measurement and a standardized Z-score, increasing the precision of risk estimation. Fourth, the study employed multiple analytical approaches, including subgroup analyses, sensitivity testing, RCS and GAM analyses, to thoroughly assess the consistency and linearity of the associations. Lastly, the observation that the UA–NAFLD relationship was more pronounced in specific subgroups suggests potential biological heterogeneity. These innovations build upon the current literature and provide a more refined framework for categorizing patient profiles and tailoring therapeutic approaches for individuals with T2DM.

Although this research benefits from notable advantages—such as a representative participant pool and rigorous statistical evaluation—certain constraints remain. Primarily, the cross-sectional nature of the study precludes establishing any definitive cause–effect link between elevated serum UA levels and NAFLD cannot be established. The observed associations merely reflect correlations, and prospective cohort studies are needed to explore potential causal or mechanistic links. Second, NAFLD identification in this study relied upon imaging of the abdomen via ultrasonography, which is widely accepted in large-scale epidemiological studies but lacks the sensitivity of liver biopsy in detecting disease severity, such as NASH or advanced fibrosis. Third, although multiple metabolic confounders were adjusted for, residual confounding remains possible. Factors such as dietary habits, physical activity levels, genetic predisposition, or individual differences in UA excretion were not measured and may have influenced the results. In addition, this investigation was based on data from a retrospective analysis performed in a single institution, a general hospital located in central China, which could restrict the extent to which the results are applicable to wider or more heterogeneous populations. Finally, key mechanistic biomarkers—including inflammatory markers, indicators of insulin resistance, and liver fibrosis scores—were not available, restricting deeper investigation into the pathophysiological role of UA in NAFLD development. Future research should focus on multicenter, longitudinal studies incorporating more comprehensive metabolic and inflammatory profiling, aiming to clarify how UA contributes to NAFLD development and to determine whether it could serve as a therapeutic target.

## 5 Conclusions

In summary, the present findings indicate that elevated serum UA concentrations and the condition of hyperuricemia are each independently correlated to a heightened likelihood of NAFLD in patients with T2DM, suggesting their potential utility in clinical screening and risk assessment. Considering the substantial occurrence rate and notable health impact of NAFLD in diabetic populations, routine monitoring of UA could facilitate prompt recognition of individuals at elevated risk and promote tailored approaches to disease management. Looking ahead, the integration of UA and related metabolic indicators into predictive models—leveraging tools such as artificial intelligence and clinical decision support systems—may enhance early detection strategies and promote more targeted interventions for NAFLD among patients with T2DM.

## Data Availability

The raw data supporting the conclusions of this article will be made available by the authors, without undue reservation.
